# Do current approaches to assessing therapy related adverse events align with the needs of long-term cancer patients and survivors?

**DOI:** 10.1186/s40959-018-0031-4

**Published:** 2018-06-15

**Authors:** Syril D. Pettit, Rebecca Kirch

**Affiliations:** 1 Gillings School of Global Public Health, University of North Carolina, Chapel Hill, NC USA; 2 Health and Environmental Sciences Institute, Washington DC, USA; 3 National Patient Advocate Foundation, Washington DC, USA

**Keywords:** Survivorship, Adverse effects, Cancer therapy, Patient reported outcomes

## Abstract

The increasing efficacy of cancer therapeutics means that the timespan of cancer therapy administration is undergoing a transition to increasingly long-term settings. Unfortunately, chronic therapy-related adverse health events are an unintended, but not infrequent, outcome of these life-saving therapies. Historically, the cardio-oncology field has evolved as retrospective effort to understand the scope, mechanisms, and impact of treatment-related toxicities that were already impacting patients. This review explores whether current systemic approaches to detecting, reporting, tracking, and communicating AEs are better positioned to provide more proactive or concurrent information to mitigate the impact of AE’s on patient health and quality of life. Because the existing tools and frameworks for capturing these effects are not specific to cardiology, this study looks broadly at the landscape of approaches and assumptions. This review finds evidence of increasing focus on the provision of actionable information to support long-term health and quality of life for survivors and those on chronic therapy. However, the current means to assess and support the impact of this burden on patients and the healthcare system are often of limited relevance for an increasingly long-lived survivor and patient population.

**Electronic supplementary material:**

The online version of this article (10.1186/s40959-018-0031-4) contains supplementary material, which is available to authorized users.

## Background

The evolution of the field of cardio-oncology is emblematic of an increasing awareness of the scope and impact of cancer therapy-related cardiac toxicities and a desire to limit these effects in both current and future patients. The literature is extant with study of the unintended and delayed but potentially severe cardiac effects of anthracyclines (as used for decades to treat some childhood cancers, breast cancer, etc.) [1–7]. Unfortunately, even for novel therapeutic approaches such as checkpoint inhibiting immunotherapy, the community again finds itself facing unanticipated and uncertain cardiac adverse events (AE) associated with the primary therapy [8, 9]. This phenomenon begs the questions of whether the cardio-oncology community specifically and cancer care community broadly (research, drug development, regulatory review, clinical practice, patients and their advocates) have improved in their ability to anticipate and support treatment-related AEs that may manifest in long-term cancer survivors.

The nexus of increasing therapeutic efficacy and the reality that 40% of the population will be diagnosed with cancer in their lifetime creates an important new public health challenge [10, 11]. The timespan of cancer therapy administration, care, and outcome is shifting from primarily acute treatment settings to a broad range of chronic adjuvant therapy and survivorship settings [12–14]. Treatment-related AEs may span in severity from potentially lethal cardiotoxicities to less dire but still debilitating systemic events, including fatigue, gastrointestinal issues, skin inflammation, and neuropathy [15–21]. The cumulative effect of these outcomes can vary considerably from patient to patient. AEs can inhibit the curative value of a therapy if these effects impede a patient’s ability or willingness to continue therapy [22]. Modulatory factors such as variable treatment adherence rates, drug-drug interactions, access to care, and patient comorbidities result in a range of patient experiences and healthcare system demands [23]. Even for a specific treatment-related AE (e.g., treatment of aromatase inhibitor–induced chronic pain or approaches to monitoring ejection fraction changes associated with anthracycline cardiotoxicity), the nature of the supportive approaches that are adopted may vary considerably from site to site [24, 25].

**Fig. 1:**
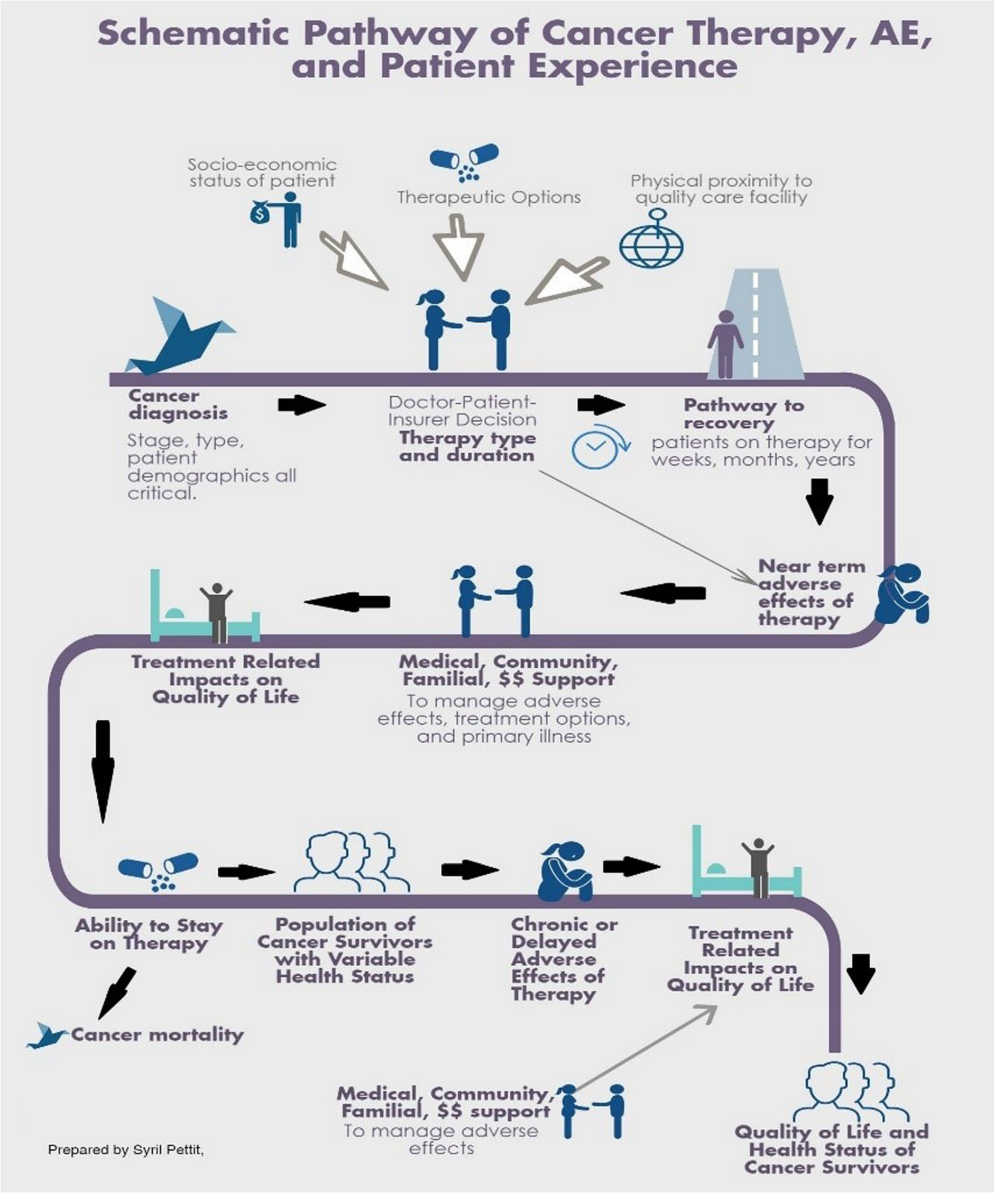
Schematic pathway linking cancer treatment, survivorship, adverse events, and quality of life (Original figure by Syril Pettit)

Observational studies and health record analyses demonstrate that cancer treatment-related AE (both moderate and severe) can also degrade patients’ or survivors’ overall health status, cause financial strain, and limit their ability to meet family obligations, work, or pursue fitness or hobbies [12, 23]. Supportive care to ameliorate AEs may require patients to procure a broad range of pharmacologic treatments, undergo monitoring and testing, change diet and exercise practices, seek out rehabilitation services or pursue complementary alternative medicine approaches like acupuncture [26–30]. In sum, the impact of AEs on long-term cancer patient and survivor quality of life are broad and diverse in their manifestation (Fig. 1).

Ideally, of course, our ability to predict, limit, support, and/or prevent cardiac and other AE associated with cancer therapy would evolve in parallel with therapy development and approval. In practice, however, the growth of the cardio-oncology field has been a function of the many medical epidemiology and cohort studies, practitioner case reports, and innovative translational and mechanistic studies initiated *only after many decades of observing the profound manifestation of these toxicities as morbidity and mortality in cancer survivors*. The cancer care community has an opportunity and obligation to transition towards a more proactive stance on the collection and use of AE information to improve patient outcomes and quality of life.

In order to support the growing population of survivors and patients receiving chronic cancer therapy, a broad set of stakeholders (including drug developers, regulators, insurers, clinicians, patients and their families) will require actionable and integrated risk and benefit information to support long-term health and quality of life. However, a successful transition towards greater proactivity and broadened time horizons for such assessments will require more than an aspiration. It will depend heavily on the consistent use of relevant metrics and robust data integration frameworks.

This paper explores comprehensive literature reviews to evaluate some of the contemporary systemic measures and frameworks used to assess and communicate potential AEs and their impact on patients. Because the systematic approaches to detecting, reporting, integrating, and acting upon on cancer therapy AEs are neither limited to nor unique for cardiovascular AEs – these reviews consider a breadth of endpoints in their discussions and analyses. The specific relevance of these approaches for aiding cardiologists, oncologists, and others in providing support to patients and survivors will be addressed.

## Data sources and drivers

Before any oncology drug moves into clinical practice in the United States, the balance between acceptable AEs (risk) and efficacy (benefit) is influenced and assessed by pharmacologic drug design, nonclinical testing, clinical trials, and regulatory review, all of which are major investments spread across the private and public sectors. During this process, the risk:benefit ratio for the therapy is calibrated (by the drug developer and regulatory reviewers) against the lethality of the target cancer [31]. Broadly speaking, these approaches provide an accepted and protective means of balancing anticipated AEs with efficacy in the patient population [32]. Interindividual variability in response to treatment, heterogenous tumor types, and limited study durations have, however, been cited as challenges in the generation of population-level and/or individual patient-level biological outcomes [33]. As a result, investment in enhancing preclinical predictivity is a significant area of growth. The biomedical research community is pursuing the adoption of novel preclinical experimental platforms, innovative preclinical and clinical trial designs, the use of comparative effectiveness methods, and enhanced collection of patient-reported AE data to enhance the predictive relevance of premarket safety and efficacy data [34–38]. It is clear, however, that the current effort and investment in nonclinical oncology safety studies and clinical trials generates data that are more highly focused on and predictive of some outcomes (e.g., acute organ toxicities) than others (e.g., chronic pain or delayed onset events such as cardiac damage) [38, 39].

On the other end of the drug development horizon, and at the genesis of the field of cardio-oncology, are data and experiences from patients outside of a clinical trial or nonclinical study setting. This arena is now often referred to as the source for ‘real world evidence’ (RWE). RWE can be defined as *“*information on health care that is derived from multiple sources outside typical clinical research settings, including electronic health records (EHRs), claims and billing data, product and disease registries, and data gathered through personal devices and health applications” [40]*.* The cancer community at large is actively exploring opportunities to leverage this type of approach across broad cancer therapy classes and patient demographics. These efforts seek to use RWE in relation to a marketed drug or set of therapeutic approaches to promote a “learning healthcare system” (LHS) in the United States [40–42]. The LHS concept, initiated by the Institute of Medicine in the early 2000s, promotes the generation of “the best evidence and to apply that evidence to the healthcare choices that each patient and provider make in collaboration; to drive the process of discovery as a natural outgrowth of patient care; and to ensure innovation, quality, safety, and value in health care.” [43]

Although RWE has been instrumental as a primary evidence base for delayed cardiac effects of cancer therapy, its feasibility as an operational means to realize a more iterative and interconnected healthcare system at large is less clear. The means to integrate RWE as a complement to regulatory safety evaluation via randomized controlled trials (RCTs) and/or as a means of generating novel efficacy, safety, or use information for marketed drugs remains uncertain [40–42]. For example, Sherman et al. (2017) cite the potential for RWE (e.g., postmarket surveillance or postmarket trials) to help refine dose-setting, subpopulation identification, and long-term safety considerations for novel cancer therapeutics that receive expedited initial approval. The model they describe, however, does not truly expand the traditional approach to drug evaluation and retains the longstanding emphasis on standard safety/efficacy endpoint collection and regulator-mediated evaluation and decision making. Novel clinical trial designs and settings (e.g., the National Institutes of Health Collaboratory), large-scale health record analysis (e.g., Million Veterans Program), and new patient-engaged networks (e.g., PCORnet) have also been cited as potential opportunities to generate RWE [44–46]. As these are all relatively new programs (less than 3 years), their impacts are not yet defined. Ultimately, the success and novelty of any RWE approach to informing healthcare will rely upon the generation of credible, fit for purpose, and otherwise unavailable information as well as viable channels to disseminate and use this information. If indeed RWE is intended to enhance the value of health data (AE-driven or otherwise) to a range of stakeholders, a more nuanced and diverse evaluation of stakeholder needs will be required.

To this end, approaches to better engage patients as the primary stakeholder abound. The movement to provide “patient-centered care” with “shared decision making” is in part driven by a desire to enhance therapeutic adherence and efficacy by engaging patients with understandable information about the benefits, risks, costs, and logistics of their treatment [47–52]. This too is a challenging space as, not unexpectedly, preferences for balance of QoL versus length of life vary from patient to patient (and can vary during the course of therapy) [53, 54]. As such, the measurable impact of new patient-centered interventions is an area of active study with regard to the role of patient satisfaction on therapeutic adherence and health outcome [55, 56]. Although the concepts of patient-centered care are widely embraced conceptually, the way in which these elements are pragmatically incorporated into practical care decisions or data generation incentives remains loosely defined [52].

## Aligning information and patient need

Despite both the conceptual objectives described in the prior section and the considerable practical experiences in the cardio-oncology arena, to date, there has been minimal systematic assessment of the alignment of available integrative approaches in the published literature with the demographic and temporal realities of an ever-growing cancer survivor population. This review focuses on the source data and assumptions relating to the use of AEs as part of integrative evaluations used to inform therapy-related decisions and patient care for long term treatment or survivorship settings. As the value of these approaches will depend upon their flexibility and external validity, this study includes but will not be limited to cardio-oncology specific applications.

## Approach to reviewing the literature

The discussions below reflect the integration of two distinct but complementary comprehensive literature reviews on the use of AE data in characterizing the impact of cancer therapy on long-term cancer patients and survivors. The first review focuses on economic modeling approaches as these were the primary format for integrated “valuation” of the impact of treatment-related AEs before the year 2016. This publication is the first systematic evaluation of the variance in these methodologies with a specific emphasis on their underlying assumptions and data sources. The review evaluates the diversity of AEs and costs evaluated, the range of populations studied, and the relevance of these metrics towards informing and supporting patient quality of life. The second review moves forward in time to recognize the 2016 release of novel integrated ‘value frameworks’ by several professional societies including American Society of Clinical Oncology and the European Society of Medical Oncology. These frameworks were proposed as novel, integrative tools to combine toxicity, efficacy, cost, and other factors deemed critical for treatment and supportive care decision-making. The second literature review identifies the ways in which the recent flurry of integrative value frameworks has (or has not) improved on pre-2016 approaches and the likelihood that these frameworks will support enhanced access to actionable information for patients and clinicians.

Specifically, the reviews address two foundational approaches in the context of long-term cancer therapy and survivorship:

- *Review 1:* How have economic models been designed and populated to measure the impact of treatment-related AEs on cancer patients and the healthcare system?
- *Review 2:* What recommendations have been promoted to improve the quality and/or relevance of AE-related input data for cancer care value frameworks?

The results of each literature assessment follow below individually and then are integrated to define common approaches, key strengths and limitations, and consensus recommendations for future needs. The specific relevance to the cardio-oncology arena is addressed.

## Review 1: Cost as a proxy for AE impact – Integrative assessments before 2016

Over the last 10 years, quantitative efforts to capture the *impact* of treatment-related adverse events have primarily taken the form of economic studies and quality-adjusted life-year (QALY) models and projections These efforts are largely aimed at economic, regulatory, and/or policy audiences. The few available estimates of the cost of purchasing and administering cancer therapy, monitoring health while on therapy or after, and managing AE detection and care, point to a major societal investment—as much as $120 billion and growing annually [23, 57]. In the cardio-oncology arena, the cumulative direct costs of supportive care for delayed cardiac effects are difficult to source. However, several studies have looked at the cost effectiveness of interventions such as pre-emptive left ventricular ejection fraction screening and determined that it is likely to be a cost-effective measure towards detecting dysfunction before it translates into heart failure [58].

Almost irrespective of the cost figures themselves, the exercise of estimating ‘total’ cost associated with the long-term effects of therapy allows for a thoughtful examination of a range of clinical, lifestyle, financial, social, and temporal elements that extend well beyond the scope of the typical U.S.-based drug safety assessment. In the U.S., the Food and Drug Administration (FDA) is not mandated to consider financial impacts when making regulatory approval decisions for oncology drugs and thus does not consider cost factors in its decision making although such approaches are routinely used in evaluating drugs in Europe [59, 60]. The use of cost as a means of capturing the totality of treatment-related AEs on patients is an approach subject to some debate in economic, clinical, patient, and medical ethics communities [61, 62]. For example, in the context of anthracycline induced cardiac toxicity, the ‘value’ of dexrazoxane as a cardio-protective agent has been an ongoing focus for studies seeking to align data around its protective effects, safety, cost, and societal willingness to pay [63–66]. Despite the sometimes conflicting outcomes, these studies have a vital role in informing an evolving landscape of methods that integrate diverse data of relevance for characterizing long-term cancer patient care and support.

A 2013 review covered some of these topics as they related to studies between 1999 and 2009 with a primary focus on whether QoL, multiple dose administration, and multiple AEs were considered in the cost assessment [67]. We have extended this review by incorporating material from the years 2007–2017, enhancing the focus on the source of AE data and AE terminology (ontology), characterizing the target patient population to whom the cost/risk predictions apply, and exploring assumptions around the cost of AEs and related supportive services. Although these issues span across the globe, for purposes of limiting the healthcare delivery context referenced in this analysis, the discussions here are focused primarily on the United States. Specifically, the review below asks: *How have economic models been designed and populated to measure the impact of treatment-related AEs on cancer patients and the healthcare system?*

### Search strategy

A semi-systematic search of articles between 2007 and 2017 was conducted using the following databases: PubMed, Web of Science, CINAHL Plus with Full Text, and EconLit. Additional studies were identified through a manual search of references in relevant articles (snowballing) and evaluation of resources from leading organizations in the cancer care arena in the United States (e.g., ASCO). This research focused on those studies which specifically seek to characterize the costs (economic, social, logistical) of managing and treating adverse effects of oncologic therapy. The search terms, exclusion criteria, and Preferred Reporting Items for Systematic Reviews and Meta-Analyses [PRISMA] diagram detailing the literature procurement, filtering, and review strategy are available as online supplemental materials (Additional file 1: Table S1-Table S2, Additional file 2: Figure S1). After comprehensive search, 631 unique citations were identified, 49 were deemed eligible for full text review, and 27 were deemed fit for this analysis. A summary of key findings follows below.

## Results

The results of this structured review (2007–2017) provide insight into both the procedural means and situational assumptions around defining the costs of AEs associated with oncologic therapy [68–94]. The studies covered a broad range of therapeutic drug classes, cancer types, and patient populations. The major methodologies used in the 27 studies reviewed were: a) mathematical modeling (Markov models) using historical data and assumptions around the probability that a hypothetical patient would move across different states of disease, health, and death at various points in their care, b) estimations of the hypothetical total cost of care in support of AE based on compilations of diverse sets and sources of data, c) prospective collection of AE incidence, treatment, and cost information for actual patients, or d) meta-analyses of other published cost studies. The studies employed one of two general approaches: estimation of the total cost of a therapeutic regime (drug costs, clinical visit costs, adverse effect costs, etc.) or assessment of the cost of one or more specific AEs associated with a designated cancer therapy. The methods for representation of the cost assessment varied across the studies and included calculation of additional quality adjusted life years (QALY)s relative to total treatment cost, incremental cost to avoid a particular AE, total accumulated costs during a given treatment period (primary treatment costs and AE-related costs), total accumulated costs to treat AE only, and costs per progression-free life-year (PFLY).

The authors looked across the 27 studies to assess their respective approaches assessing and sourcing cost, adverse event, quality of life, and patient demographic information. The specific treatment of these issues in each of the 27 articles reviewed for this study is available as Additional file 1: Table S3**-**S7. The analysis demonstrated that, despite the differences in the approaches and focus across these articles, several consistent themes and trends were evident (Table 1). As will be discussed further in Results, these themes point to systemic limitations in the availability of patient relevant and externally valid data sources and approaches.

**Table 1:** Common themes and conclusions identified in review of literature on AE cost determination

Key Observations from Literature Review on AE Cost Determination	
**Frequent use of modeled and patchworked datasets.** AE cost estimation studies relied on modeled data, assumptions about patient experience, and/or data pooled from diverse sources and studies in 96% of studies reviewed.	
**Reliance on randomized control trials (RCT) as source of data on frequency and nature of AEs in patient population.** RCTs from Phase II, III, and/or IV clinical trials served as the primary data source for frequency and nature of the AEs in these cost studies (~ 70% of studies). A small percentage (19%) of studies used ‘postmarket’ databases such as the Premier Perspective Database (e.g., data from 600 U.S. hospitals) as a resource to identify the frequency and nature of AEs requiring clinical care.	
**Limited consideration of non-severe adverse events.** For cost estimation studies utilizing clinical trials, the vast majority incorporated only those AEs that were reported as severe (grade 3 or grade 4). *Note: Consensus Toxicity Criteria for Adverse Events (CTC-AE), includes a standardized list of outcomes and symptoms in oncology trials and includes a severity grading scale associated with these effects* [93]*. Grade 1 is the least severe and can include outcomes like fatigue. Grade 4 indicates very severe toxicities (like liver failure), and grade 5 denotes death associated with an adverse treatment effect.*	
**Near absence of contemporary patient-reported data on quality of life impacts.** Only one of the 27 studies (4%) identified in this review incorporated direct measures of QoL into the cost assessment [71] via surveys of participating patients. About half of the math modeling studies used “utility factors” to incorporate QoL-related adjustments. These adjustment factors appear to have been based primarily on EuroQol 5D surveys and time trade-off (TTO) surveys conducted in prior clinical most of which were conducted in the early 2000s. Discussion of the relevance of the utility factors selected was minimal to absent.	
**Absent or internally inconsistent demographic information.** The relationship between the target patient demographics (e.g., age, gender, race, geography, etc.) and the demographic from whom the cost or AE incidence data was derived was missing/or incongruous in ~ 80% of studies reviewed.	
**Limited consideration of patient-relevant indirect costs.** Incorporation of elements such as lost wages for time off work, caregiver costs, and lost future employment potential were only incorporated in 25% of the reviewed studies. An exclusive focus on direct costs (defined as cost of a hospital or physician visit associated with a treatment-related adverse event) in modeled or cumulative cost estimates was noted in~ 75% of studies reviewed.	

## Review 2: 2016 value frameworks – Better tools for Cancer care decision making?

Beginning in 2016, the practice of integrative therapy impact assessment took a significant conceptual step forward with the release of five major “value frameworks.” Value frameworks were designed to inform policy decisions as well as pragmatic therapy choices *by clinicians and patients* for a broad range of cancers, patient demographics, and therapy classes. They aim to integrate data on efficacy, safety (AEs), patient QoL, and, in some cases, cost for specific therapeutic modalities [95–99]. Specifically, the 2016 frameworks and their self-proclaimed objectives are as follows:

- *American Society of Clinical Oncology (ASCO) Value Framework*: “A framework that would enable a physician and patient to assess the value of a particular cancer treatment regimen given the patient’s individual preferences and circumstances” [95]*.*
- *European Society for Medical Oncology’s (ESMO) Magnitude of Clinical Benefit Scale (MCBS)*: “The ESMO-MCBS is an important first step to the critical public policy issue of value in cancer care, helping to frame the appropriate use of limited public and personal resources to deliver cost effective and affordable cancer care” [100].
- *Institute for Clinical and Economic Review (ICER) Value Assessment Framework*: “Ultimately, the purpose of the value framework is to form the backbone of rigorous, transparent evidence reports that, within a broader mechanism of stakeholder and public engagement, will help the United States evolve toward a health care system that provides sustainable access to high-value care for all patients” [98].
- *Memorial Sloan Kettering Cancer Center’s (MSKCC) DrugAbacus*: “DrugAbacus provides a way of thinking about how to price drugs. This interactive tool takes more than 50 cancer drugs and lets you compare the company’s price to one based on value” [97].
- *National Comprehensive Cancer Center Network (NCCN) Evidence Blocks*: “The goal is to provide the health care provider and the patient information to make informed choices when selecting systemic therapies based upon measures related to treatment, supporting data, and cost” [101]*.*

A novel comparative summary of the frameworks with respect to incorporation of AE and QoL specifically is provided here (Table 2**).** It is notable that AE/toxicity data (typically from published clinical trial data) are incorporated in all of the frameworks as a means of characterizing this key aspect of treatment choice.

**Table 2:** Comparison of five major value frameworks regarding the use of toxicity and adverse event approaches

Framework	Objective	Efficacy & safety data sources	Scoring/output	Efficacy/safety-related input data
ASCO	Inform joint decision making by patients and clinicians	Clinical trials	• Generates a single composite scored called the ‘Net Health Benefit’ (NHB) • Uses different algorithms for advanced disease vs adjuvant setting	• Uses AE data drawn from clinical trials. • Can incorporate adjustments for QoL, treatment-free interval, improvement in cancer symptoms. Can score for disease free survival (cure) or progression free survival.
ESMO	Inform public policy, clinical guidelines, and direct clinical care	Clinical trials	• Semi-quantitative process results in assignment of letter score (A–C) for adjuvant setting • Semi-quantitative process results in assignment of number score (1–5) for advanced disease	• Can score for disease free survival (cure) or progression free survival. • “Toxicity” and QoL rating incorporated.
NCCN	Inform joint decision making by patients and clinicians	Clinical trial and expert opinion	• Assigns a series of Evidence Block Scores (5-point high score, 1-point low score) categories such as toxicity, efficacy, cost, etc.	• Incorporates a range of both qualitative and quantitative inputs that are qualitatively synthesized via expert panels.
ICER	Provide synthesis for use by policymakers and payers/formularies	Clinical trials, econometric studies	• Compares standard intervention and new treatment relative to short term costs and longer-term healthcare system burdens and benefits.	• Includes quality-adjusted life year scoring factors. • Serious AEs are factored into scoring • Ability to work while on therapy factored into scoring
DrugAbacus	Provide pricing data for use by policymakers and payers	Drug safety /efficacy data as provided to FDA	• Factors benefits and burdens of treatment into a new “price” based on Abacus algorithm relative to industry-specified price.	• Scores improved survival rate • Serious AEs (e.g., grade 3 or greater) incorporated into scoring • The probability that a patient discontinues treatment because of toxicity is considered in scoring • Treatment novelty, R&D cost, health burden and treatment duration

Table 2 was modified from tables previously published in Chandra et al. (2016), Cohen, Anderson, & Neumann (2017), and Schnipper & Bastian (2016). AE, adverse event; ASCO, American Society of Clinical Oncology; DFS, disease-free survival; ESMO, European Society for Medical Oncology; FDA, U.S. Food and Drug Administration; ICER, Institute for Clinical and Economic Review; NCCN, National Comprehensive Cancer Network; NHB, net health benefit; PFS, progression-free survival; QALY, quality-adjusted life-year; QoL, quality of life; R&D, research and development. See Definition section for explanation of terms

Although the construct of these five frameworks and their intended audiences have been compared previously, this study further characterizes the current and future utility of these frameworks with respect to integration of AEs and patient reported outcome (PRO) information [102–106]. Specifically, this review addressed the following: *What recommendations have been promoted to improve the quality and/or relevance of AE-related input data for value frameworks?*

### Search strategy

A systematic literature search was conducted using the following databases: PubMed, Web of Science, and CINAHL Plus with Full Text. Additional studies were identified through a manual search of references in relevant articles (snowballing). The search terms, exclusion criteria, and PRISMA diagram detailing the literature procurement and review strategy are available as Additional file 1: Tables S8-S9, and Additional file 3: Figure S2.

### Results

This review captures recommendations and perspectives from a total of 17 peer-reviewed publications [95, 102–104, 106–118]. The reviewed studies were almost evenly split between those including a narrative/qualitative comparison of different frameworks and those incorporating case study/quantitative comparisons across different value frameworks. With limited exceptions, all studies provided perspectives on opportunities to improve either the construct of the framework or some aspect of the input data.

The most commonly identified areas for improvement, in relation to the nature and quality of input data used to populate these frameworks, were divided into eight categories per Table 3. No one recommendation or modification to improve the relevance of the frameworks for informing patient QoL was cited by all the publications. However, the need for more robust and/or detailed safety and toxicity data inclusion in these frameworks was the most common recommendation identified.

**Table 3:** Literature-based Recommendations for Improvement of Inputs to Existing Frameworks

Suggested improvement	No of Articles	References
Need improvements to clinical trial design to obtain more patient-relevant data	5 of 17 (29%)	[102, 110, 112, 115, 119]
Need cost data that reflect full cost of care/treatment (not just drug costs)	5 of 17 (29%)	[104, 108, 112, 116, 117]
Frameworks should incorporate patient-reported outcome data (via inclusion of patient-reported outcomes in clinical trials)	4 of 17 (24%)	[103, 112, 119, 138]
Frameworks should incorporate data from sources other than clinical trials (e.g., observational studies)	3 of 17 (18%)	[102]
Frameworks should incorporate more robust and/or detailed safety and/or toxicity data	7 of 17 (41%)	[103, 104, 108, 112, 115, 116]
Frameworks should use integrated quality of life measures in lieu of safety data	1 of 17 (6%)	[117]
Frameworks should incorporate more longitudinal data	2 of 17 (12%)	[102, 103]
Frameworks should engage patients in the data evaluation and input process	3 of 17 (18%)	[102, 103, 110]

Additionally, several studies called for more overarching changes to clinical trial design with regard to patient inclusion criteria, duration, outcomes measures, and so forth [102, 110, 112, 115, 119]. The details of such modifications were not thoroughly addressed in these publications and are the subject of much discussion elsewhere, but they could have significant impact on the type of AE data generated in the future [120].

Although not the focus of this review, it is important to note that many of the publications also called for broad-based improvements in the design or use of the frameworks themselves. Specifically, enhanced clarity and transparency as to the intended audience for the framework outputs [103, 104, 110, 111, 121] and improved guidance to enhance reproducibility were common recommendations [107, 108, 111, 114, 119, 122].

## Limitations of these reviews

These reviews have a number of limitations. Because this study sought to assess impact in a U.S. healthcare context, economically based health technology assessments (HTAs) as required in Europe and several other regions to assess the cost-benefit of novel therapies were not incorporated. HTA studies are numerous and relatively standardized in their approaches and assumptions. Although HTAs relate only to single-payer healthcare systems that do not match the current U.S. multi-payer profile, they could provide potentially useful sources for methodological comparisons. Because selected HTAs also include quality of life (QALY) assessment in their economic evaluation of the cost-benefit of the therapy they can also provide a resource in this regard for financial valuation-focused queries.

Similarly, the term “comparative effectiveness” was intentionally excluded in order to exclude the “comparative effectiveness research” literature. Although these studies do sometimes include cost estimates of AEs, their focus is exclusively on the differential/comparator between two similar therapies and thus the total cost of AEs (the focus of this review) is rarely measured [67]. Prior systematic reviews of AE effect cost assessments have noted this limitation in the use of comparative effectiveness studies and thus they were excluded from this search. This review also did not compare the absolute value of reported costs across studies because of the variable drugs, study designs, timescales, and patient populations assessed.

Additionally, the breadth of U.S.-based studies in this review provides an opportunity to characterize a diverse range of methods, but it also means that comparison across studies at a granular level is limited. Future studies might focus on a single drug class or cost assessment approach to allow for more focused cross-study comparison of input data and conclusions. Additionally, more comprehensive insights into methodological and data input assumptions across these studies could be gleaned by review of key underlying studies cited by the studies reviewed here.

## Synthesis of results of literature evaluations

Characterizing the risk:benefit profile of an antineoplastic therapy requires integration of a complex and heterogenous mix of pharmacologic, economic, actuarial, ethical, and sociologic factors. The complementary literature searches described here illustrate progress toward this integration. However, with respect to use and integration of AE information, several common themes and areas for improvement were identified. These broad areas of commonality and their relevance to the field of cardio-oncology are discussed in detail below.

### Challenges in use of clinician-reported adverse event data derived from randomized controlled trials

In both the purely economic and integrated value framework approaches reviewed here, Phase II–III RCTs serve as the primary source of data on the incidence of treatment-related adverse effects. Almost 70% of the cost studies reviewed here (and even more of the value frameworks) rely heavily or exclusively on RCT data to inform toxicity/safety. RCTs are accepted, well-controlled studies with defined inclusion criteria and dosing and monitoring strategies. However, many of the studies reviewed here noted the limitations of RCTs for purposes of providing pragmatic patient decision support (i.e., high internal validity but low external validity). These limitations include the following:

- ***Population Mismatch.*** Populations engaged in RCTs tend to be “healthier” and with fewer comorbidities than the average patient population on the therapy [123]. The frequency and severity of AEs in the clinical trial population may be under-representative of AE incidence and severity in the broader patient population and may thus lead to an underestimation of overall cost and incidence burden [124].
- ***Severity Skew.*** The type of AEs recorded in Phase II/III trials specifically have been reported to skew toward a focus on only high-grade (grade 3 or 4) toxicities, pool toxicities of varying severity, include both quantitative and qualitative evaluations, and/or misgrade toxicities [125, 126]. Thus, it is possible that a significant pool of AE data may be systematically excluded from these evaluations. Even when lower-grade toxicities are reported in RCTs, this review demonstrates an almost exclusive (~ 80%) use of the high-grade AE data for purposes of cost modeling or in value frameworks. The ASCO framework was recently revised to allow for incorporation of grade 1 and 2 AEs if they occur at sufficient frequency [95]. Given the tendency to under-report low-grade AEs in trials and published concerns about “unclear reporting of lower-grade toxicities,” the potential for these endpoints to usefully inform patient/clinician choice via integrative tools is limited at present [127–129].
- ***Limited Duration.*** The timeframe of study in an RCT provides a limited window (months to ~ 4 years) for capture of treatment-related effects. This limitation is of particular concern in the cardio-oncology arena as many relevant cardiac AEs do not manifest for many years after the closure of therapy and/or persist for many years after therapy has been completed. As such, RCTs may provide an incomplete picture of impact.
- ***Uncertain Naming Conventions.*** The evolving nature of the CTCAE ontology used to record and grade AEs creates a “moving target.” The number of terms has expanded by a factor of 4 in the last 20 years. Thus, the version of CTCAE (or other ontology) can have a significant impact on the nature, naming, and overall reported incidence of AEs used in cost evaluation studies. Several clinical specialty areas that address common antineoplastic treatment-related AEs (e.g., rheumatology) have developed their own AE ontologies and grades to reflect the more nuanced perspective of a specialist [130]. Experts have noted that the recording of AEs by non-specialists can result in non-specific calls such as ‘cardiac-general’ with no detail about the nature of the cardiotoxicity [127]. Without additional and well-sourced granularity in these toxicity reports, it will be challenging for regulators, clinicians, and patients to appropriately calibrate expectations and supportive care needs. Future AE incidence burden evaluations would benefit from a thorough characterization of the ontological and inclusion/exclusion framework that guided the capture of their core input data and its potential impact on outcomes.
- ***Repurposing.*** RCTs are designed for evaluation by regulatory scientists for purposes of drug approval decision making. Although they are routinely used in this manner, these studies are not specifically designed to generate information to be used by clinicians or patients with regard to individual therapeutic or supportive care pathways.

### Alternatives to the use of clinician-reported adverse event data derived from randomized controlled trials

**Fig. 2:**
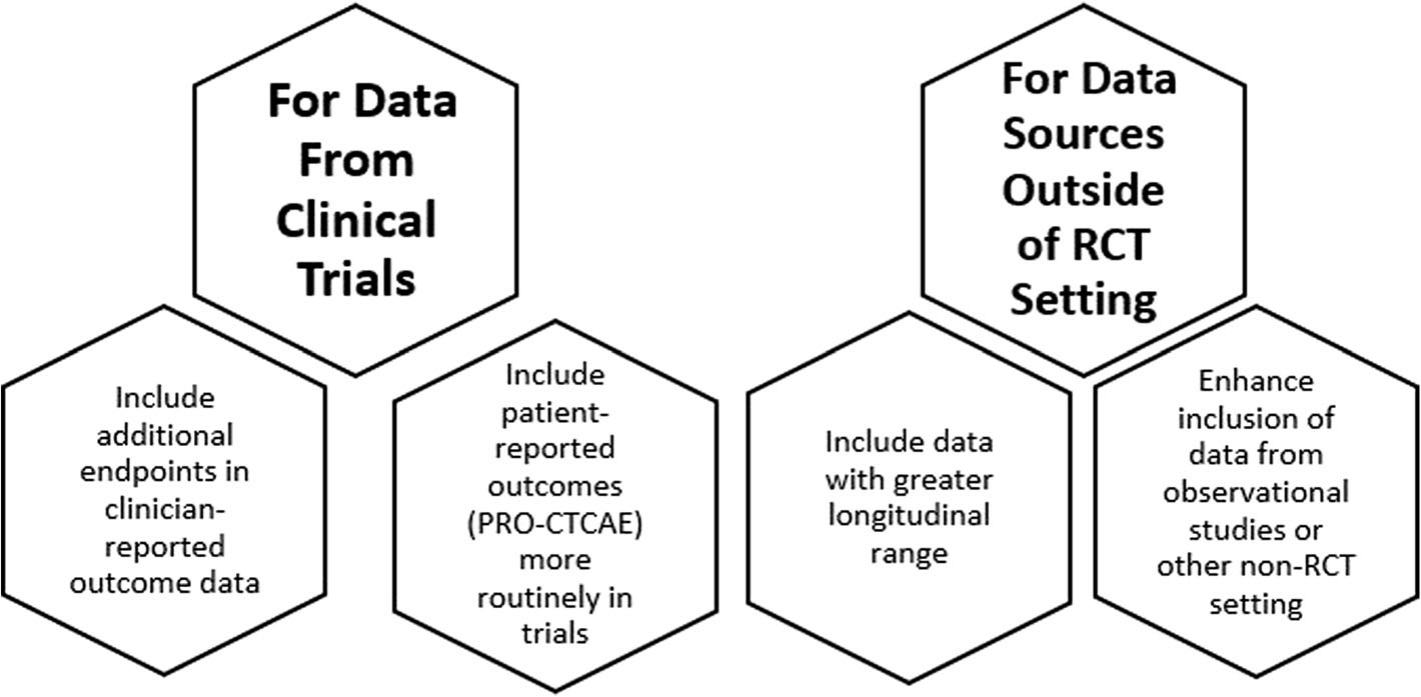
Summary of recommendations from literature review for improving adverse event data relevance in value frameworks

Although of many of the studies reviewed here identified one or more shortcomings of RCT-derived AE data, only three (17%) of the publications on value frameworks proposed the future incorporation of data from sources outside of an RCT setting [102–104]. Similarly, only 18% of the cost models used data sources outside of RCTs for AEs [72, 74, 75, 87, 88]. This trend points to a simple fact: while it is relatively easy to identify weaknesses in the RCT as a data source for informing patient value-choices, the identification of viable alternatives or complements is quite challenging. As illustrated in Fig. 2, the articles reviewed here identified potential opportunities for improvement via 1) increased use of PRO measures; and/or 2) increased use of observational/surveillance/EHR data sets*.*

### Patient-reported outcomes and quality of life metrics

As patients and clinicians seek both enhanced progression-free survival as well as positive QoL, the inclusion of PRO data and/or QoL metrics into the valuation (economic or otherwise) of antineoplastic therapy regimes has gained prominence in recent years. The sources of data on QoL in the studies in this review included Markov model-based utility factors derived from EuroQol 5-D surveys, direct patient surveys that collected data on quality metrics, and clinician judgement on impact of patient QoL. The ICER and ESMO frameworks include QoL through incorporation of a QALY metric. ASCO uses palliation of symptoms and treatment-free intervals as a proxy for QoL measures. However, the relevance of these input data are uncertain, as some of the QoL adjustment factors used in these economic evaluations were derived from assessments conducted as many as 20 years ago and some included undocumented “value judgements” based on clinician experience [77, 90, 99, 131]. Additionally, the way in which these data were integrated into the value assessments described in this review varied from probabilistic modeling approaches to awarding of ad hoc “bonus points.” It is beyond the scope of this review to assess the relative strengths and weakness of each of these approaches. However, this review does reveal that the practice of including PRO or QoL metrics into integrated value assessments relating AEs and treatment choice is neither systematic nor standardized.

Undoubtedly, this is a challenging space. The tools and perspectives on the extent to which QoL or PROs can or should be leveraged routinely in trials or clinical practice are evolving rapidly [132]. We also know that there are often disparities between patient and clinical perspectives on the scope and nature of AE burden [133]. Thus, the future use of tools to assess AEs from the perspective of the patient may provide novel insights into the overall physical, logistical, and financial burden of antineoplastic therapy. As patients or survivors experiencing cardiac AEs associated with their curative therapy are likely to experience impacts on their ability to work, exercise, engage in recreation, etc., QoL considerations are expected to be of significant relevance to therapy adherence and outcome.

### Increased use of observational/surveillance/electronic health record data sets

Collectively, the publications reviewed here offered very limited recommendations for or examples of incorporating AE data from sources other than RCTs. The few prospective or patient-database driven economic evaluation studies in this review appear to provide a clearer picture of the frequency and nature of AEs, although the less controlled setting can make an estimation of treatment-attributable costs more challenging [72, 74, 75, 87, 88]. None of the value frameworks utilize non-RCT data this time. This phenomenon reflects the “gold standard” status of RCTs for driving drug safety and efficacy decisions and lack of standards for use of other data sources. Increasingly, the potential for observational studies and large-scale healthcare databases to provide reliable data on a broad range of patient adherence practices, outcomes measures, and polypharmacy/comorbidity situations has been recognized [134–136]. Future developments in this arena will require a thoughtful confrontation of the tension between uncontrolled data derived directly from patient care settings and the value of nuanced and realistic representation of patient experiences.

### The costs

A detailed discussion of cost estimation methodology is not the focus of this review. However, the link between value decisions, cost calculations, and AE-related impacts is an important component of this discussion. Some economic evaluation studies reviewed here attempted to include all treatment-related costs that the author could identify (drug cost, hospital cost, doctor visits, monitoring and testing, over-the-counter drugs, administration fees, lost work cost, caregiver costs, future employment potential costs, etc.; e.g., Sorensen et al., 2012), whereas others addressed only the primary cost of treating the AE in a hospital setting [72]. Cost metrics used within the current value framework structures were equally variable but are largely restricted to cost of the drug and/or primary treatment visits. Many of the analyses reviewed here specifically recommended that future iterations of these models should incorporate the full cost of care including AEs [104, 108, 116, 117, 137] This recommendation, while sound on its face, begs the questions of what constitutes the burden of antineoplastic therapy-related AEs, who carries these burdens, and how broadly should costs be captured. It also speaks to the importance of transparent discussion regarding the stakeholders to whom the value assessment is intended to apply.

### The patients

Patients are at the core of all of the value discussions and treatment choices described here. Surprisingly, nearly a third of the economic valuation studies reviewed here failed to provide clear demographic information on either the patient population that constituted their input data or the patient population to whom their model/valuation was intended to characterize or both [80, 82, 86, 89, 100, 111, 114]. In fact, none of the primary value frameworks described in Table 2 or any of the publications about these frameworks (as reviewed here) included a discussion of patient demographics *other* than a focus on patients with a specific disease. Even for those studies where the patient population was clearly defined, there were sometimes disconnects between the target population and the patient group that served as primary data on AEs, QoL metrics, and/or cost estimations. For example, ~ 40% of the studies reviewed here utilized cost data from Medicare, although only ~ 20% characterized their study population as older than age 60. Such disconnects may be inevitable given the limited availability of data in this space. However, the relevance of frameworks for informing patient choice and treatment decisions could be enhanced with greater clarity around these limitations and their potential impacts on the way in which AE-related impacts are synthesized and subsequently interpreted.

## Improving the use of AEs to inform assessments of treatment impact on patients & survivors

We began these reviews by questioning whether current data capture and integration systems are improving in their ability to anticipate and proactively support AEs such as treatment-related cardiotoxicity. On the one hand, these reviews reveal a growing focus on the interests of patients, payers, clinicians, regulators, and drug developers in making informed choices around the use of antineoplastic therapies that enhance progression-free survival with minimal impact on overall QoL. However, it is also clear that our current means to assess and synthesize the scale and impact of this burden on patients, survivors, and the system at large remains insufficient in many ways. Much of the input data used in current integrative efforts to describe AE incidence and severity is of limited or unclear relevance for the diverse, long-lived, and comorbid patient population that is rapidly growing around the world. We observed an overall lack of clarity around how to best use AE data to inform cancer patient care and cancer therapy safety assessment – particularly in long term cancer therapy and survivorship contexts of relevance to the cardio-oncology field. This uncertainty suggests that we may continue to struggle with the proactive identification of treatment-related cardiac (as well as other) effects that take years to fully manifest or to be potentiated. Indeed, our ability to effectively capture the scope and impact of relatively rapid onset (but not highly severe) AEs appears limited as well.

Moving forward, limitations around the data, as well as the implications of those limitations in terms of how they may affect the patient’s lived experience and quality of life should be made more evident to patients, clinicians, and other stakeholders when possible. This communication is essential in supporting more person-centered cancer care planning and shared decision making that helps align treatments with patients’ values and preferences. While such information may not immediately influence treatment decisions, it can have important downstream effects. First, it can seed critical exchanges around supportive care, quality of life priorities and expectations, and health monitoring. These exchanges have the potential to positively influence patient outcomes. Second, as patients and their family caregivers become more educated about AE’s, they have the opportunity to leverage their influence as primary stakeholders to demand improvements that prioritize information quality, access to appropriate supportive care and monitoring, and AE-reducing treatment options in the future for maximizing both quantity and quality of life.

## Conclusion

The current breadth of approaches for integrating AE, QoL, efficacy, and cost can be viewed as a signal of the public health and cancer communities’ commitment to and investment in these issues. However, the continued reinvention of these approaches also suggests that the complement of current efforts may not be adequately synergistic or fit for purpose. While the development of the cardio-oncology field has offered some important visibility to the impact of treatment-related AE on patient survival and QoL, it does not appear to be associated with any broad systemic transformations in AE-relevant data collection or use. The biomedical community has made incredible progress in treatments resulting in step-changes in progression free survival. However, the biomedical and public health communities have not adequately transitioned to AE-related information collection and use frameworks that fully aligns with this increasing therapeutic efficacy and survivorship. Future research and associated action is needed to ensure that understandable and reliable information about long term outcomes can be made more relevant and accessible for a growing population of long-term survivors and the stakeholders who seek to support them.

## Additional files

Additional file 1: **Table S1**. Search terms for literature review. **Table S2**. Inclusion and exclusion criteria. **Table S3**. Cost assessment methodologies. **Table S4**. Defining costs. **Table S5**. Nature/frequency of treatment-related adverse events. **Table S6**. Incorporation of “quality of life”. **Table S7**. Defining the population. **Table S8**. Search strategy. **Table S9**. Inclusion/exclusion criteria. (DOCX 138 kb)

Additional file 2: **Figure S1** PRISMA diagram demonstrating the part I literature evaluation and exclusion process. (JPG 100 kb)

Additional file 3: **Figure S2** PRISMA diagram demonstrating the part II literature evaluation and exclusion process. (JPG 72 kb)
