# Supplementary material for: Do current approaches to assessing therapy related adverse events align with the needs of long-term cancer patients and survivors?
Source: Cardiooncology. 2018 Jun 15;4:5. doi: 10.1186/s40959-018-0031-4 (PMC7048033; doi:10.1186/s40959-018-0031-4)
Supplement: Supplementary file 1 — Table S1. Search terms for literature review. Table S2. Inclusion and exclusion criteria. Table S3. Cost assessment methodologies. Table S4. Defining costs. Table S5. Nature/frequency of treatment-related adverse events. Table S6. Incorporation of “quality of life”. Table S7. Defining the population. Table S8. Search strategy. Table S9. Inclusion/exclusion criteria. (DOCX 138 kb) [file 40959_2018_31_MOESM1_ESM.docx]

SUPPLEMENTAL TABLES

Table S1: Search terms for literature review

| **Date Limitations: 2007 to Present** | |
| --- | --- |
| **Concept** | **Key Words, Search Terms** |
| Cancer focus | “**c**ancer” or “oncology” |
| AND | |
| Treatment | “therapy” or “treatment” or “therapies” or “drugs” or “medication” |
|  | AND |
| Adverse effects from therapy | “safety” or “toxicity” or “adverse effect” or “adverse event” or “toxicities” or “harm” |
|  | AND |
| Cost and/or burden evaluation | “value of treatment" or “cost of toxicity” or “cost of toxicities” or “cost of adverse effects” or “burden of toxicity” or “burden of toxicities” or “burden of adverse effect” or "toxicity management"  or “cost-effectiveness” or “pharmacoeconomics” |

Table S2: Inclusion and exclusion criteria

| **Inclusion criteria** | **Exclusion criteria** |
| --- | --- |
| - Qualitative and quantitative studies | - Focus of study is exclusively or primarily on healthcare delivery outside of the United States |
| - Descriptive and analytical studies | - Studies that do not substantively evaluate cost or treatment burdens associated with adverse consequences of cancer treatment |
| - Methodological and meta-analyses that employ primary analysis of data | - Article is older than 2007 |
| - Focused primarily on the U.S. healthcare system. Studies that included non-U.S. system evaluation were also included as long as U.S. healthcare evaluation was also incorporated | - Studies focused on the cost of “best supportive care” defined as provision of palliative care in the absence of an antineoplastic regimen [139] |
| - Studies that measure adverse events associated with a prescribed pharmacologic intervention to treat cancer | - Studies not in English |
|  | - Chemoprevention or homeopathic studies |
|  | - Studies for which the intervention is surgical or radiological |
|  | - Summary reports or reviews unless they include novel analyses |

Table S3: Cost assessment methodologies

| **Topic** | **Number of articles** | **References** |
| --- | --- | --- |
| Studies utilizing Markov models to estimate cost  *(Note: Markov models are widely used for health economic analyses to evaluate potential outcomes of a disease process. The model reflects movement across different health states that are predefined by the modeler.)* | 11/27 (40%) | [67,71,73,74,84,86,89,90,92] |
| Studies conducting retrospective cost estimation based on analysis of published data from trials or other sources | 14/27 (52%) | [67,68,71,73,74,76,77,84–86,89,90,92] |
| Studies conducting prospective data collection and cost estimation | 1/27 (4%) | [72] |
| Meta-analyses across published cost studies | 1/27 (4%) | [77] |

Table S4: Defining costs

| **Topic** | **Number of articles** | **References** |
| --- | --- | --- |
| Studies incorporating “indirect” costs such as time off work, caregiver costs, or lost employment potential | 6/27 (22%) | [67,72,76,80,81,87] |
| Studies utilizing Medicare as a proxy for cost data for drugs and services required to treat an adverse event | 11/27 (41%) | [67–72,74,78,79,85,86] |
| Studies utilizing prior peer-reviewed studies as a proxy for cost data for drugs and services required to treat an adverse event | 6/27 (22%) | [76,77,83,84,92] |
| Studies utilizing private healthcare or state healthcare costs to estimate fees regarding adverse events | 7/27 (26%) | [80–82,87,88,90,91] |
| Studies using the Healthcare Cost and Utilization Project to estimate costs associated with adverse events | 4/27 (15%) | [73,75,89,93] |

Table S5: Nature/frequency of treatment-related adverse events

| **Topic** | **Number of articles** | **References** |
| --- | --- | --- |
| Studies using postmarket databases to support adverse event type and incidence**;** includes electronic health records, surveillance studies, cohort studies, etc. | 5/27 (19%) | [78,80,88,90,91] |
| Studies citing other sources of data on adverse event type and incidence | 2/27 (7%) | Direct patient survey and case reports: [72]  Drug label data: [81] |
| Studies utilizing RCTs as a primary source of data on incidence and nature of AE | 19/27 (70%) | [67–71,73,75–77,79,82–87,89,92,93] |
| *One study w AE data from an unclear source -* [74] |  |  |

Table S6: Incorporation of “quality of life”

| **Topic** | **Number of articles** | **References** |
| --- | --- | --- |
| Studies incorporating quality of life via the use of **“**utility factors**”** derived from prior literature | 9/27 (33%) | [67,69,71,73,82–84,87,93] |
| Studies incorporating measures of quality of life based on de novo measures/assessments by the investigator | 1/27 (4%) | [72] |

Table S7: Defining the population

| **Topic** | **Number of articles** | **References** |
| --- | --- | --- |
| Defines target population of the model/analysis as a patients age 60 or older | 6/27 (22%) | [67,71,73,78,83,90] |
| Studies modeling/describing patient populations of various ages | 13/27 (48%) | [68–70,72,74,77,79–81,88,91] |
| Studies in which study population/relevant population ages are not described | 9/27 (33%) | [75,82,84–87,89,92,93] |

Table S8: Search strategy

| **No date limitations: search conducted July 2, 2017** | |
| --- | --- |
| **Concept** | **Key words, search terms** |
| Cancer Focus | “Cancer” or “oncology” |
| AND | |
| Value frameworks | “value framework” |
|  | AND |
| Adverse effects from therapy | “safety” or “toxicity” or “adverse effect” or “adverse event” or “toxicities” |

Table S9: Inclusion/exclusion criteria

| **Inclusion criteria** | **Exclusion criteria** |
| --- | --- |
| - Studies that provide analysis or assessment of one or more comprehensive “value framework(s)” or an equivalent approach to integrate adverse event evaluation into an overall pharmacologic intervention to treat cancer | - Primary/seminal framework references were excluded |
| - Qualitative and quantitative studies | - Studies not using adverse event and/or safety data to assess cancer treatment interventions and decision making |
| - Descriptive and analytical studies | - Article is older than 2012 |
| - Peer-reviewed studies | - Studies not in English |
|  | - Studies relating to frameworks for assessing risk to environmental carcinogens or exposures |
|  | - Meeting reports and non–peer-reviewed studies |
