# Supplementary figures and images for: Do current approaches to assessing therapy related adverse events align with the needs of long-term cancer patients and survivors?
Source: Cardiooncology. 2018 Jun 15;4:5. doi: 10.1186/s40959-018-0031-4 (PMC7048033; doi:10.1186/s40959-018-0031-4)

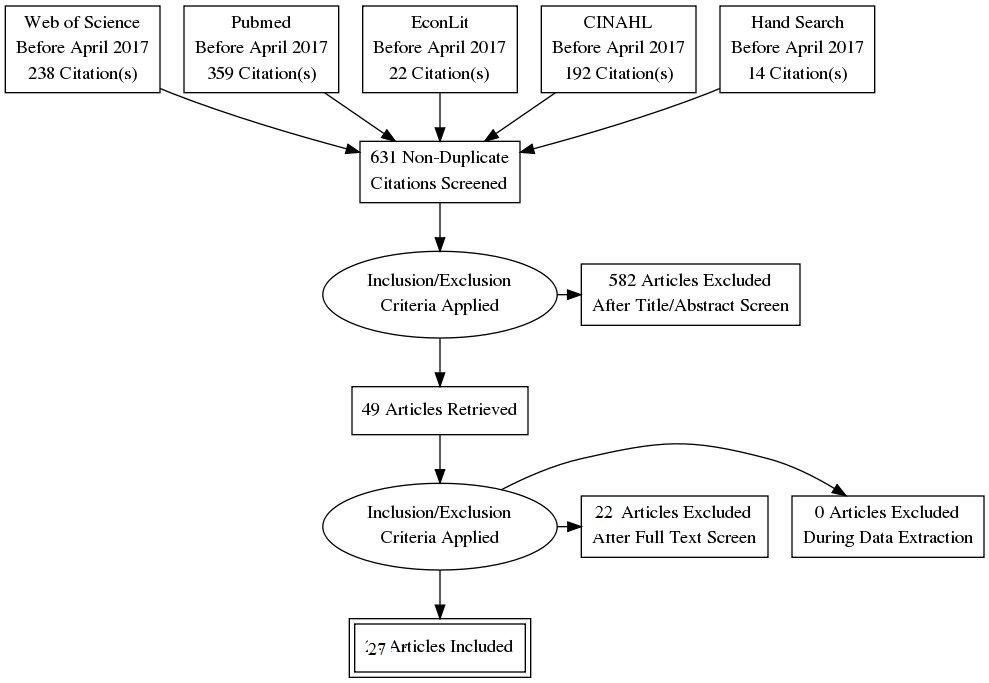

Supplement: Supplementary file 2 — Figure S1 PRISMA diagram demonstrating the part I literature evaluation and exclusion process. (JPG 100 kb) [file 40959_2018_31_MOESM2_ESM.jpg]

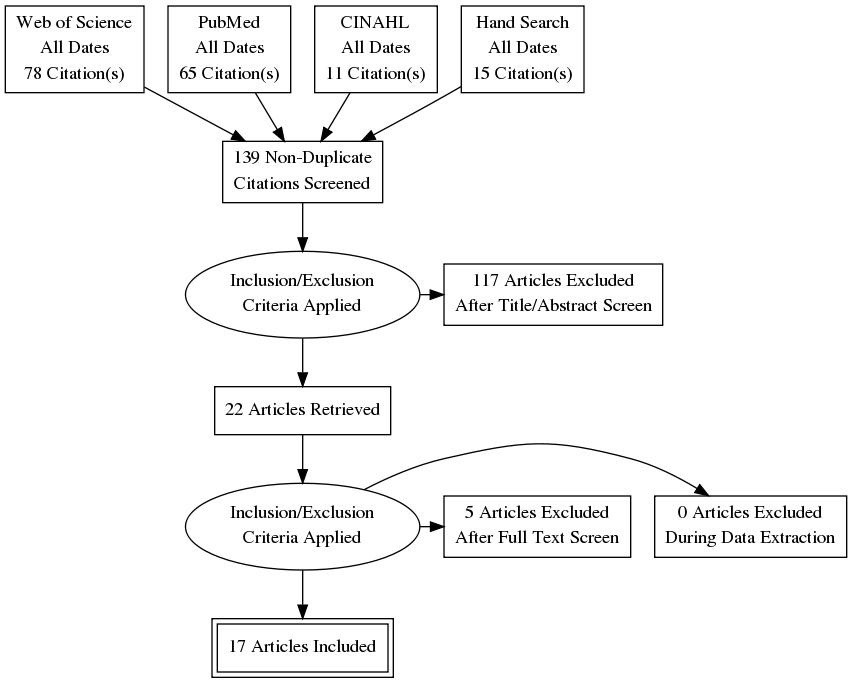

Supplement: Supplementary file 3 — Figure S2 PRISMA diagram demonstrating the part II literature evaluation and exclusion process. (JPG 72 kb) [file 40959_2018_31_MOESM3_ESM.jpg]
